# Microvesicles-hydrogel breaks the cycle of cellular senescence by improving mitochondrial function to treat osteoarthritis

**DOI:** 10.1186/s12951-023-02211-8

**Published:** 2023-11-15

**Authors:** Senrui Liu, Shengwen Cheng, Bowen Chen, Pengcheng Xiao, Jingdi Zhan, Jiacheng Liu, Zhuolin Chen, Junyan Liu, Tao Zhang, Yiting Lei, Wei Huang

**Affiliations:** Department of Orthopaedic Surgery, The First Affiliated Hospital of Chongqing Medical University, Chongqing, 400016 People’s Republic of China

**Keywords:** Cellular senescence, Mitochondrial dysfunction, Stem cells, Responsive hydrogel, Microvesicles

## Abstract

**Background:**

Osteoarthritis (OA) is an age-related disease characterised by the accumulation of senescent chondrocytes, which drives its pathogenesis and progression. Senescent cells exhibit distinct features, including mitochondrial dysfunction and the excessive accumulation and release of reactive oxygen species (ROS), which are highly correlated and lead to a vicious cycle of increasing senescent cells. Stem cell therapy has proven effective in addressing cellular senescence, however, it still has issues such as immune rejection and ethical concerns. Microvesicles (MVs) constitute the primary mechanism through which stem cell therapy exerts its effects, offering a cell-free approach that circumvents these risks and has excellent anti-ageing potential. Nonetheless, MVs have a short in vivo half-life, and their secretion composition varies considerably under diverse conditions. This study aims to address these issues by constructing a ROS-responsive hydrogel loaded with pre-stimulant MVs. Through responding to ROS levels this hydrogel intelligently releases MVs, and enhancing mitochondrial function in chondrocytes to improving cellular senescence.

**Result:**

We employed Interferon-gamma (IFN-γ) as a stem cell-specific stimulus to generate IFN-γ-microvesicles (iMVs) with enhanced anti-ageing effects. Simultaneously, we developed a ROS-responsive carrier utilising 3-aminophenylboronic acid (APBA)-modified silk fibroin (SF) and polyvinyl alcohol (PVA). This carrier served to protect MVs, prolong longevity, and facilitate intelligent release. In vitro experiments demonstrated that the Hydrogel@iMVs effectively mitigated cell senescence, improved mitochondrial function, and enhanced cellular antioxidant capacity. In vivo experiments further substantiated the anti-ageing capabilities of the Hydrogel@iMVs.

**Conclusion:**

The effect of MVs can be significantly enhanced by appropriate pre-stimulation and constructing a suitable carrier. Therefore, we have developed a ROS-responsive hydrogel containing IFN-γ pre-stimulated iMVs to target the characteristics of ageing chondrocytes in OA for therapeutic purposes. Overall, this novel approach effectively improving mitochondrial dysfunction by regulating the balance between mitochondrial fission and fusion, and the accumulation of reactive oxygen species was reduced, finally, alleviates cellular senescence, offering a promising therapeutic strategy for OA.

**Supplementary Information:**

The online version contains supplementary material available at 10.1186/s12951-023-02211-8.

## Introduction

Population ageing has become a global issue of increasing significance, accompanied by a rise of age-related diseases [1]. As individuals age, the functions of their tissues and organs gradually decline, encompassing various systems of the body. Osteoarthritis (OA) is a degenerative condition characterised by joint pain and dysfunction, and research reveals that approximately 27% of individuals aged 60 years and above exhibit imaging evidence of knee osteoarthritis, with this percentage increasing with age [2, 3]. Numerous studies indicate that cellular senescence plays a crucial role in the development of age-related diseases, even though the commonalities and processes of various age-related diseases remain incompletely understood [4]. The onset of OA is closely related to the accumulation of senescent cartilage cells. Cellular senescence is typically defined as an irreversible cessation of the cell cycle due to replicative stress [5]. Although macrophages can clear senescent when present in small numbers, the accumulation rate of senescent cells gradually increases with age and various stimuli, eventually exceeding the rate of clearance [6].

In addition to cell cycle arrest, senescent cells exhibit various characteristics, including heightened levels of increased reactive oxygen species (ROS) and mitochondrial dysfunction, which are highly correlated [7]. Under normal conditions, cells generate low levels of ROS for signalling purposes. However, as cells enter a state of ageing due to age or various stimuli, mitochondrial function weakens, resulting in reduced membrane potential, increased proton leakage, continual ROS accumulation, and impaired ATP production [8, 9]. Elevated ROS levels further induce the sustained release of numerous harmful cytokines, known as the senescence-associated secretory phenotype (SASP) [10, 11]. These SASP components, in conjunction with excess ROS, are released into the extracellular environment, elevating local ROS concentrations in the microenvironment. This not only prompts adjacent normal cells to enter a state of senescence, but also exacerbates mitochondrial damage, ultimately leading to a vicious cycle of cellular senescence [12]. Recent research has indicated that preserving mitochondrial membranes potential holds promise in slowing or even reversing the ageing process. Restoring mitochondrial function and morphology can lower ROS generation [13]. Therefore, the restoration of mitochondrial function has become an important potential approach in anti-ageing therapy [14].

Stem cell therapy is a method employed to facilitate tissue regeneration and repair by introducing stem cells into the patient's body. Among these, mesenchymal stem cells (MSCs) have gained substantial attention due to their potential in anti-oxidative stress and anti-ageing [15, 16]. However, this form of stem cell therapy faces ethical and safety challenges, notably the risk of tumour formation and immune rejection [17, 18]. Moreover, research has revealed that the primary mechanism through which stem cells yield therapeutic benefits does not entail direct tissue differentiation and replacement but is instead mediated through paracrine pathways [19]. Therefore, the microvesicles (MVs) released by MSCs measuring 100 nm to 1 μm in diameter, are recognised as crucial components in the success of stem cell treatments [20, 21]. These MVs harbour rich bioactive molecules, including miRNAs, proteins, and cytokines, capable of regulating tissue physiology and metabolism, mitigating oxidative stress, stimulating tissue regeneration, and ameliorating cell ageing by regulating mitochondrial energy metabolism [22]. Furthermore, their nanoscale size enhances their ability to penetrate challenging tissues, such as the cartilage matrix, reaching depths that conventional stem cell therapy cannot attain [23]. Consequently, MVs offer reduced risk, enhanced biological safety, and significant potential for improving cell senescence and energy metabolism balance, making them as an emerging, dependable, and safer option for anti-ageing therapy [24, 25].

On the other hand, the immunomodulatory activity and content of MVs released by stem cells exhibit significant variability under different stimulus conditions. Inflammatory mediators serve as a “trigger” to elicit a defensive response from MSCs, and the MVs released at these moments function as endogenous “immunomodulators”. Diverse types of MVs generated in response to distinct stimuli have demonstrated superior performance in targeting various diseases [26–28]. Interferon-gamma (IFN-γ) is a significant cytokine in senescent cells, which plays a pivotal role for both inducing cellular senescence-like phenotypes and eliminating senescent cells; therefore, we have selected it as the trigger for stem cell senescence therapy leading to the creation of specific IFN-γ-microvesicles (iMVs) [29–31]. Nonetheless, regardless of the type of MSCs MVs employed, challenges persist, including their short half-life and susceptibility to external interference, which limit their clinical application and effectiveness [32]. Hence, the development of an efficient delivery system for stem cell MVs has emerged as a prominent area of research. This delivery system can help transport MSCs MVs securely to the treatment site, minimising loss and inactivation, and ultimately enhancing their therapeutic effect [33, 34].

**Fig. 1:**
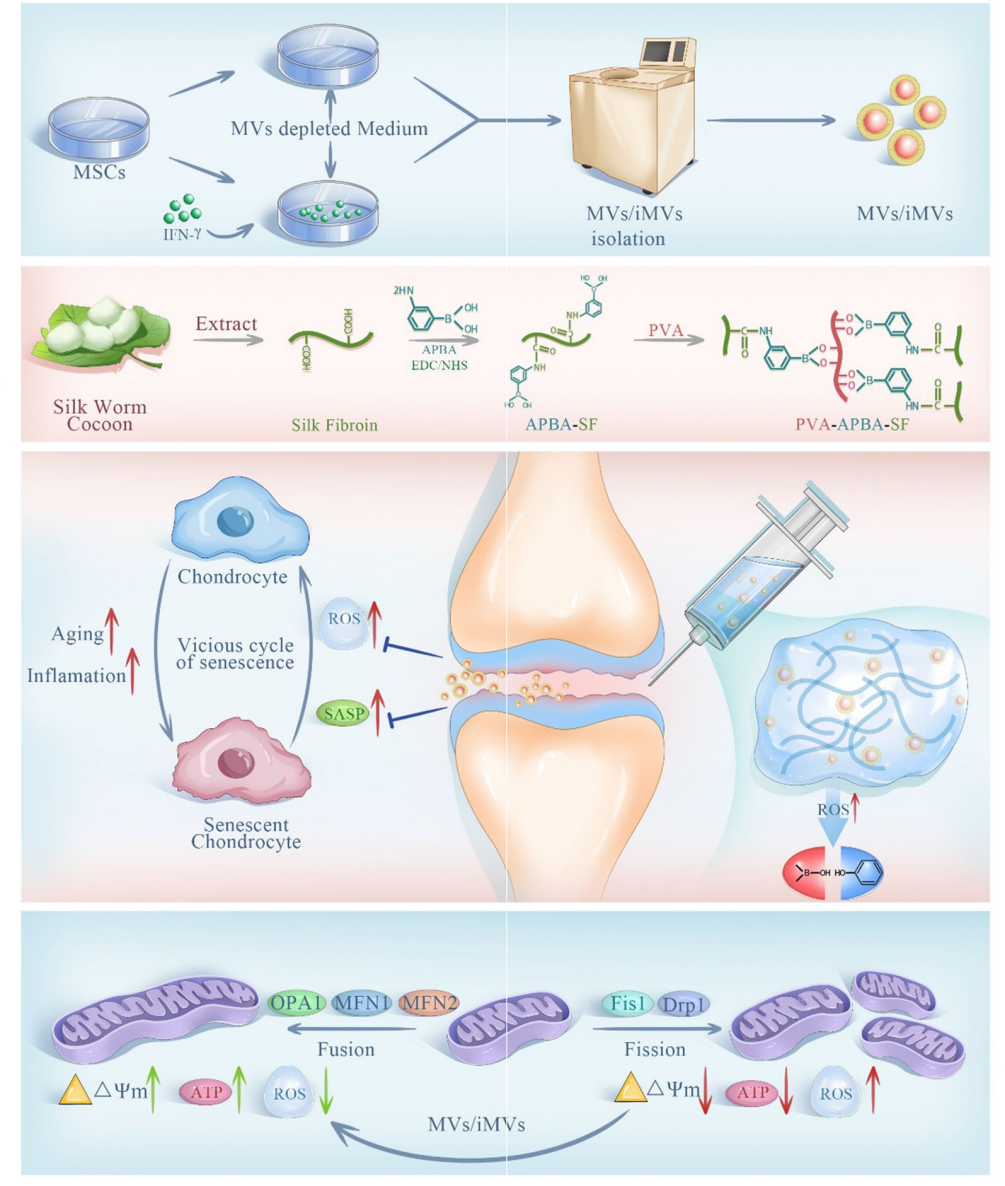
The principle and production of anti-ageing Hydrogels@MVs/iMVs. MVs and iMVs can be obtained by different interventions and separated by gradient centrifugation. SF is extracted from natural silk cocoons, synthesized into a hydrogel with responsive functionality, and used as a carrier for MVs/iMVs. After injection into the joint cavity, they can exert a therapeutic effect on chondrocytes. Inhibiting excessive mitochondrial fission in chondrocytes and promoting mitochondrial fusion can prevent the vicious cycle of chondrocyte senescence caused by ROS

Highly biocompatible and intelligently sensitive hydrogels are gaining prominence in tissue engineering and serving as versatile drug delivery vehicles [35–37]. These responsive hydrogels exhibit the ability to react to both physical and chemical factors within and outside the body [37, 38]. On one hand, they serve as a protective shield for medications during administration, safeguarding them from the influence of physiological conditions. On the other hand, they can precisely govern the rate and timing of drug release, thereby improving treatment outcomes [39–41]. Since local inflammation microenvironments in ageing tissues display substantially increased ROS and decreased pH, and the heightened local ROS levels primarily result from the vicious cycle of chondrocyte ageing, we have developed a novel hydrogel designed to be responsive, enabling the intelligent release of MSCs MVs. In this work, we used an ROS-responsive hydrogel as a vehicle for MSCs MVs by grafting 3-aminophenylboronic acid (APBA) onto silk fibroin (SF) and simply combining it with polyvinyl alcohol (PVA). Notably, in vitro experiments have demonstrated that this newly created PVA-APBA-SF hydrogel can extend the functional duration of MVs to 20 days, while remaining highly responsive to ROS. Moreover, this synthetic hydrogel possesses qualities such as injectability and self-healing. By safeguarding the MVs and prolonging their half-life, they can be continuously and intelligently released to more effectively counteract cell ageing and disrupt the vicious cycle of ROS-mediated ageing. To assess the effectiveness of this material and select the appropriate MVs, weconducted evaluations on the carrier material’s ability to combat the vicious cycle of ageing mediated by continuous ROS increase. We also compared the effects of the standard Hydrogel@MVs with Hydrogel@iMVs concerning chondrocyte ageing, energy metabolism, and mitochondrial motility. These results provided novel approaches to combat of cell ageing (Fig. 1).

## Materials and methods

### Materials

Silkworm cocoons were generously provided by the Chongqing Sericulture Science and Technology Research Institute, located in Chongqing, China. The following materials were procured from Macklin Co.: N-(3-dimethylaminopropyl)-n′-ethylcarbodiimide hydrochloride (EDC), N-hydroxysuccinimide (NHS), polyvinyl alcohol (PVA; Molecular weight: 195,000), lithium bromide, and 2-mercaptoethanol (2-ME) (Shanghai, China). Additionally, we sourced Dulbecco's modified eagle medium (DMEM) with high glucose, phosphate buffered saline (PBS) and penicillin–streptomycin (5000 U/mL) from Gibco Life Technology Co. (USA). Foetal bovine serum (FBS) and MVs-depleted FBS were purchased from VivaCell Co. (Shanghai, China). All antibodies were procured from Abcam Inc. (USA). The Reactive Oxygen Species Assay Kit, Senescence-Associated β-Galactosidase (SA-β-Gal) Stain Kit and Calcein/PI Cell Viability Assay Kit were purchased from Beijing Solarbio Science & Technology Co. (Beijing, China). The BCA protein concentration kits, Enhanced ATP Assay Kit and Enhanced Mitochondrial Membrane Potential Assay Kit with JC-1) were obtained from Beyotime Biotechnology Co. (Shanghai, China). The RNA Extraction Kit was sourced from Thermo Fisher Scientific Co. (MA, USA), and the cDNA synthesis kit was acquired from MedChemExpress Co. (Shanghai, China). Both the C3H/10T1/2 cell line and ATDC5 cell line were purchased from the American Type Culture Center (Manassas, VA, USA).

### Extraction and characterisation of MSCs MVs/iMVs

To obtain two distinct types of MSCs MVs, we cultured C3H/10T1/2 cells, which are well-established mouse embryo fibroblasts serving as a mesenchymal stem cell model [42–44]. Initially, cells were seeded in 15-cm cell culture plates at a density of 5 × 10^6^ cells per dish and cultivated for 24 h in DMEM supplemented with 10% FBS, 1% penicillin–streptomycin, and maintained in a humid atmosphere with 5% CO2. For the extraction of iMVs, the addition of 10 ng/mL IFN-γ to the medium was required. Subsequently, the MV-free culture medium was utilised for both MVs and iMVs at when the cell confluence reached 50–60%. After 48 h of continued cellular secretion of MVs and iMVs, the cell supernatant was collected.

A differential centrifugation technique was employed for the isolation of MVs and iMVs [45]. In summary, the supernatant was centrifuged at 300*g* for 10 min to remove cells, followed by centrifugation at 2000*g* for 10 min to remove dead cells. Next, centrifugation at 10,000*g* for 30 min was conducted to discard cell debris. Finally, the supernatant underwent ultracentrifugation at 120,000*g* for 2 h, followed by resuspension in PBS within sterile centrifuge tubes, and temporary storage at 4 °C [21]. Subsequently, the characterisations of MVs and iMVs were evaluated, with their morphology observed using scanning electron microscopy (SEM, JEM-1200EX, Japan). The particle size of MVs and iMVs was assessed using ZetaView (Particle Metrix, Germany). Moreover, the expression of MVs surface markers CD9, CD63, and CD81 was determined through western blotting [46].

### Uptake of Exos/iExos by ATDC5

Throughout the study, ATDC5, a mouse chondrocyte line, was used [47]. ATDC5 cells were seeded in ConfocalDishes containing complete media. Isolated MVs/iMVs were stained and labelled using red fluorescent dye PKH26. After cell attachment and reaching 50% confluence, MVs/iMVs were introduced, and incubation continued for 8 h. Following fixation with 4% paraformaldehyde, the cells were labelled with phalloidin and DAPI to delineate the cytoskeleton and nucleus, respectively. The uptake of MVs/iMVs was observed by scanning the cells using a confocal laser microscope (Nikon NIE-A1plus, Japan).

### Extraction and modification of SF

The SF preparation followed previously described procedures [48]. In brief, cleaned cocoons were cut and boiled with 0.02 M Na_2_CO_3_ for 1 h, repeating this process twice to eliminate sericin. The SF was washed with distilled water and dried. A specific weight of SF was collected and dissolved in lithium bromide at 55 °C. Afterward, it was dialysed in ultrapure water for 3 days to eliminate ions and other impurities, yielding a 5 wt.% SF solution. To modify the silk protein, the SF solution was dialysed in 0.5 M MES buffer (pH = 6.0) overnight prior to the reaction. A mixture of 2 g EDC and 5.5 g NHS was stirred into a 100 mL SF solution for 30 min at room temperature to initiate the reaction. Subsequently, 1 mL of 2-ME was added to halt the reaction, followed by the slow addition of 1.5 g APBA into the solution slowly, which was stirred to dissolve, react for 2 h. After completion of the reaction, the mixture was dialysed in double-distilled water for 48 h, with water changes every 6 h. The APBA-SF solution and SF solution were freeze-dried, and Fourier analysis was conducted. Transform infrared spectroscopy (iS10 FT-IR spectrometer, USA) was employed to detect and analyse all three chemicals, including APBA, SF, and APBA-SF.

### Preparation of PVA-APBA-SF Hydrogel@MVs/iMVs

To create the PVA-APBA-SF hydrogel, MVs/iMVs suspended in PBS were initially mixed with a 5 wt.% PVA solution (molecular weight ≈ 195,000). This mixture was then diluted with double-distilled water to achieve a 2.5 wt.% concentration and allowed to rest for a specified duration. Subsequently, this solution with a 2.5 wt.% APBA-SF solution in a 3:1 ratio. This resulting mixture rapidly formed the PVA-APBA-SF Hydrogel@MVs/iMVs.

### Characterisation of hydrogel

The shape of the hydrogel was observed by employing SEM following. Additionally, laser confocal microscopy was utilised to assess the distribution of PKH26 fluorescently labelled MVs within the hydrogel, both in cross-sections and in 3D space. Dynamic rheological behaviour of the hydrogels was evaluated using a rotating rheometer (Discovery HR-2, USA). A strain scanning frequency of 1 Hzwas used, covering a strain range from 0.01 to 100% to determine the critical strain point. Furthermore, cyclic small strain (0.5%, 60 s) and large strain (50%, 60 s) tests were conducted to assess the self-healing ability of the hydrogel.

### In vitro degradation and ROS responsiveness of hydrogels

The accurately weighed hydrogel specimens were immersed in separate tubes containing PBS and H_2_O_2_ (1 mM), both supplemented with proteinase XIV to simulate the in vivo degradation environment. These tubes were continuously shaken at 37 °C. At intervals of 2 days, the hydrogel was removed, surface moisture was absorbed using filter paper, and the mass Md was recorded at different time points over a total of 21 days. Each condition at each time point had three samples. The degradation rate (percentage) was calculated using the formula (M0−Md)/M0 × 100%.

### MVs release from hydrogels

To evaluate the efficiency of exosomes release, the hydrogel was loaded into a dialysis bag (MWCO = 1000 Da) and immersed in PBS and 1 mM H_2_O_2_ respectively, while being shaken at 37 °C. Dialysis fluid was collected and replenished at regular intervals. The BCA technique was employed to quantify the MVs release efficiency by measuring the protein content in the dialysis solution [49].

### Assessment of hydrogel biocompatibility in vitro

The biocompatibility of the hydrogel was assessed using a co-culture model of hydrogel and ATDC5 cells stablished through a Transwell apparatus [50]. ATDC5 cells were cultured in the lower chamber, while Hhydrogel or Hydrogel@iMVs, along with complete growth medium, were added for cell cultivation. The control group received no hydrogel. On days 1, 3, and 5, we the hydrogel was removed, and live/dead assays were conducted to distinguish between live and dead cells. Images were captured using a fluorescent inverted microscope (Nikon Clipstie, Japan), and live and dead cells were counted. Additionally, at the same time points, cell viability was assessed through the CCK-8 assay by measuring absorbance, following the manufacturer’s instructions.

### In vitro study of the therapeutic effects of Hydrogel@MVs/iMVs on senescence in ATDC5 cells

To investigate the therapeutic effects of Hydrogel@MVs/iMVs on ageing in cartilage cells, we conducted an analysis using an SA-β-Gal stain kit. In summary, ATDC5 cells were seeded in a 6-well plate and subjected to cell ageing induction by treating them with 200 μM of H_2_O_2_ for 48 h in complete culture medium. Hydrogel@MVs/iMVs were employed in the therapy, while the control group received no H_2_O_2_ treatment. After treatment, the cells were fixed for 15 min at room temperature with 4% paraformaldehyde following two PBS washes. A premade SA-β-Gal staining solution was applied to each well, and incubated at 37 °C overnight. Subsequently, cells were washed with 70% ethanol, and after acquiring inverted microscope images, the number of positive cells in both the ageing and normal groups was counted and analysed to calculate the rate of ageing cell positivity. Furthermore, under the same treatment conditions, immunofluorescence staining of Ki67 was performed in ATDC5 cells, and images were captured using a fluorescence microscope. The proportion of Ki67-positive cells was calculated, and Western Blot experiments were conducted to assess the expression of RB protein and p-RPS6 protein in the cells, providing a comprehensive evaluation of the therapeutic effects on cell senescence [51].

### Flow cytometry

Flow cytometry was employed to assess the effects of Hydrogel@MVs/iMVs on the cell cycle of ATDC5 cells after the induction of cell senescence. The induction and grouping of cell senescence in ATDC5 cells were performed as previously described. Cells were treated, collected, fixed with 70% ethanol overnight at 4 °C, and then analysed using a flow cytometer (CytoFLEX, Beckman Coulter, Fullerton, USA).

### Examination of mitochondrial function in vitro

Cells underwent the same treatment for 48 h before being rinsed with PBS and placed in fresh complete media. Subsequently, the cells were treated for 20 min at 37 °C with the JC-1 staining working solution and observed under a fluorescence microscope. Furthermore, the cells were resuspended in JC-1 staining working solution, incubated, centrifuged, washed twice, and resuspended for analysis using a fluorescence spectrophotometer. A 490 nm excitation and 530 nm emission wavelength were used to identify JC-1 monomers, while a 525 nm excitation and 590 nm emission wavelength were employed to detect JC-1 aggregates. In order to evaluate the potential and functionality of mitochondria [52].

### ATP assessment

A suitable number of ATDC5 cells were seeded into a 6-well plate and allowed to adhere and grow until they reached 50% confluency. Senescence was induced for 48 h and then Hydrogel@MVs/iMVs were added. The control group received no treatment while the H_2_O_2_ group did not receive any hydrogel. Following the manufacturer's instructions, the culture medium was removed, and 200 μL of lysis buffer was added to each well to lyse the cells. The supernatant was obtained after centrifuging the lysate at 12,000*g* for 5 min at 4 °C. A 96-well plate was used, and ATP detection working solution and supernatant were added. Fluorescence spectrophotometry was employed to measure the ATP level in various cell types, assessing mitochondrial activity.

### Respiratory chain complex assay

After treating ATDC5 chondrocytes with H_2_O_2_, H_2_O_2_ + Hydrogel@MVs/iMVs, or control for 48 h, the cells were lysed with RIPA buffer to extract proteins for Western Blot analysis. This analysis aimed to determine the content of mitochondrial respiratory chain complexes in different groups. Protein bands were imaged and analysed using the ChemiDoc MP imaging system and ImageJ (Wayne Rasband, NIH, USA).

### Evaluation of the intracellular antioxidant impact of MVs/iMVs in vitro

Following 48 h of senescence-inducing treatment for ATDC5 cells, fresh complete medium was introduced. Then, PKH26 fluorescent dye-labelled Hydrogel@MVs/iMVs were separately added and incubated for 24 h. After the removal of the cell culture media, the DCFH-DA probe was introduced. The cells were incubated at 37 °C for 20 min in a cell culture incubator. Excess DCFH-DA that had not penetrated the cells was removed by washing them with serum-free cell culture media. An inverted fluorescent microscope was used to monitor and record the cells. ImageJ was employed to measure the fluorescence intensity of DCFH-DA to assess the antioxidant activity of the MVs.

### RT-PCR

Total RNA was isolated from each treatment group of ATDC5 cells using an RNA extraction kit and then reverse transcribed into cDNA. Real-time fluorescence quantitative PCR was used to measure mRNA expression levels. CT values (ΔΔCT) comparisons were utilised to calculate the relative levels of mRNA expression. The primer sequences (P16, P21, P53, OPA1, MFN1, MFN2, FIS1, DRP1, GAPDH) are provided in Additional file 1: Table S1.

### Osteoarthritis rat model establishment

To evaluate the therapeutic effects of Hydrogel@iMVs in vivo, we used 12-week-old male Sprague Dawley rats weighing 400 ± 20 g to establish an OA model through destabilisation of the medial meniscus (DMM) in combination with anterior cruciate ligament transection (ACLT) surgery. After 1 week of adaptation, and 3 weeks post-modelling, the OA rats were randomly divided into four groups of five rats each. The joint cavity was injected with either PBS, hydrogel, iMVs, or Hydrogel@iMVs. To ensure sterility, all rats received an intramuscular dose of penicillin (2000 μ/kg) before and after the procedure. All animal procedures were conducted under sterile conditions and were approved by the Institutional Animal Care and Use Committee (IACUC) of Chongqing Medical University.

### Histological assays

After 5 weeks of treatment, the animals were euthanised, and the knee joints were dissected. The joint tissue was fixed in 4% paraformaldehyde overnight at 4 °C and subsequently decalcified using a slow decalcification solution. Following dehydration and paraffin embedding, the tissue was sectioned in the sagittal plane (5 mm). Histological analysis was performed using haematoxylin and eosin H&E and Fast Red O-Safranin Green stains. Primary antibodies, P12 and P53, produced from rabbits, were used, and the sections were subsequently incubated with a goat anti-rabbit secondary antibody for 1 h. DAB reagent was used for staining. Additionally, the sections were stained for rabbit-derived DRP1, OPA1, MFN1, MFN2, and FIS1 using the corresponding fluorescently labelled secondary antibodies and DAPI. All staining was observed and recorded using a microscope, and quantitative analysis of relative expression levels or fluorescence intensity was conducted using Image J.

### Statistical analysis

All in vitro experiments were conducted with three biological replicates and in vivo experiments were conducted with five replicates. The data are presented as means ± standard deviations. Statistical analysis was performed using one-way analysis of variance and Tukey’s post-hoc test in GraphPad Prism (9.0). Statistical significance was defined as a P-value less than 0.05, denoted as *P < 0.05, **P < 0.01, ***P < 0.001 and ****P < 0.0001.

## Result

### Characterisation of MVs/iMVs

**Fig. 2:**
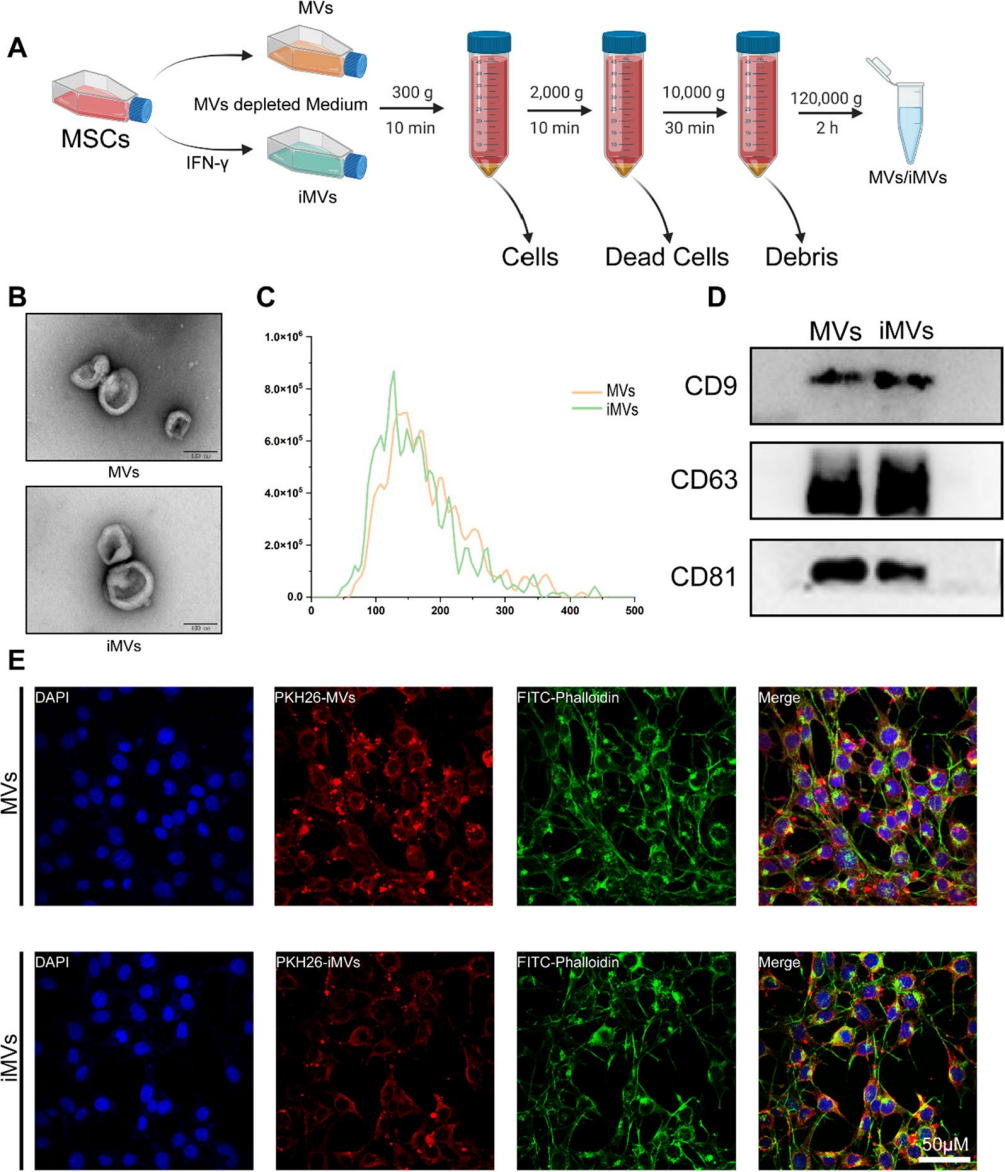
Extraction and characterization of MVs/iMVs. **A** MVs/iMVs were obtained through different intervention methods and gradient centrifugation. **B** Scanning electron microscopy images of MVs/iMVs. **C** Particle size distribution of MVs/iMVs. **D** Expression levels of surface membrane markers of MVs/iMVs were analyzed by Western blotting. **E** LSCM images of ATDC5 cells up-taken MVs/iMVs for 8 h

Following several rounds of differential centrifugation to liminate residual cells and cell debris, MVs were isolated from the supernatant using ultracentrifugation at 120,000*g* (Fig. 2A). SEM revealed that both types of MSCs-derived MVs had the characteristic double concave disc shape, with particle size analysis indicating a size range of 100–200 nm, consistent with MVs (Fig. 2B, C). We extracted MVs/iMVs proteins and analysed the expression of the membrane proteins CD9, CD63, and CD81 using Western Blotting, confirming the presence of these markers in both types of MVs (Fig. 2D). To observe the uptake of MVs by chondrocytes, we incubated MVs/iMVs labelled with PKH26 fluorescent dye in ATDC5 cell cultures for 8 h. Fluorescence confocal microscopy demonstrated that both types of MVs were effectively taken up by ATDC5 cells, with no significant differences in their characteristics, indicating their biological activity (Fig. 2E).

### Characterisation of ROS responsive hydrogel

**Fig. 3:**
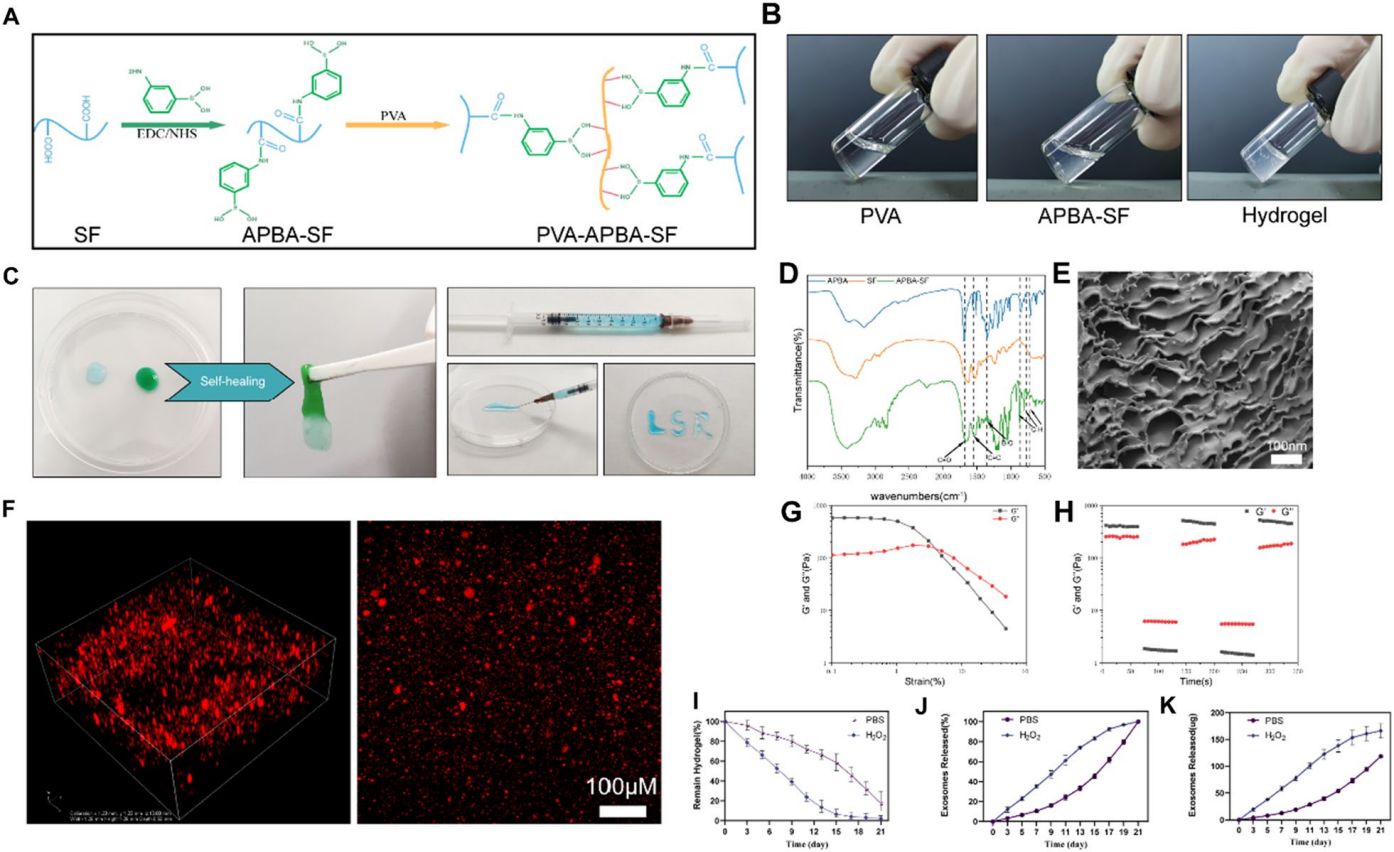
Preparation and characterization of MVs/iMVs-loaded responsive hydrogels. **A** Chemical schematic of SF modification and hydrogel synthesis. **B** PVA solution, APBA-SF solution, and resulting hydrogel formed rapidly after mixing. **C** Dynamic bonding between two separate hydrogels, injectability of hydrogel, and extrusion of hydrogel into “LSR” shape. **D** Fourier-transform infrared spectra of SF, APBA, and APBA-SF. **E** Scanning electron microscopy image of the hydrogel. **F** Confocal z-stack overlay and layer scan images of Hydrogel@MVs, with red fluorescence indicating MVs labeled with PKH26. **G** Strain sweep test of the hydrogel. **H** Continuous step-strain experiment of the hydrogel under repeated deformation at 1% and 1000% strain. **I**–**K** Degradation and release of MVs/iMVs from the hydrogel in PBS and H_2_O_2_

An ideal age-responsive material should exhibit high responsiveness to ROS, while also prolonging the half-life of MVs and protecting them from rapid degradation. Phenylboronic acid (PBA) was chosen due to its high biocompatibility and unique responsive function, making it a suitable candidate for constructing ROS-responsive materials [53–55]. In aqueous solutions, most PBA-containing polymers exist in an equilibrium state between their hydrophobic triangular form and hydrophilic tetrahedral form [56]. PBA can form reversible covalent bonds with nearby diols, making its structure more hydrophilic. When ROS levels increase, the covalent bond between PBA and boron breaks, resulting in an ROS-dependent drug release system. Additionally, ROS is consumed during the reaction, and many PBA-containing materials exhibit anti-inflammatory effects [55, 57]. SF, a natural protein offers excellent mechanical properties, high biocompatibility, low immunogenicity, and biodegradability, making it widely used in tissue engineering. SF is easy to modify and can be constructed into various biomaterials [58]. PVA, another common biomaterial, contains neighbouring diols. Thus, we catalysed the combination of APBA and SF through EDC/NHS to form an APBA-SF solution via an amide bond [52]. After mixing the APBA-SF solution with PVA, dynamic bonds were quickly formed with the neighbouring diols on PVA, leading to the formation of a gel at room temperature. This gelation process was completed within 30 s at room temperature, allowing for the creation of a hydrogel with ROS-Linker (Fig. 3A, B). We successfully injected the hydrogel using a 1-mL syringe, demonstrating its injectability. The hydrogel also has good plasticity and can be shaped as desired. We formed the letter ‘LSR’ by injection, thus demonstrating its plasticity. Due to its responsive dynamic bond, two independently separated hydrogels can re-heal together, demonstrating its self-healing property (Fig. 3C). Fourier transform infrared spectroscopy confirmed that a new peak representing the B-O bond appeared at 1340 cm^−1^ in APBA-SF. Representative substitution peaks of the benzene ring appeared at 860 cm^−1^, 810 cm^−1^, and 710 cm^−1^, confirming the successful modification with the ROS-Linker (Fig. 3D). SEM demonstrated the porous network structure of the hydrogel (Fig. 3E). To load MVs into the hydrogel, MVs were pre-mixed with PVA to ensure even distribution and then mixed with APBA-SF to form the gel. Laser scanning confocal confirmed the successful and 3D-reconstruction of red fluorescent MVs loaded onto the hydrogel indicating uniform distribution (Fig. 3F). Rheological measurements showed that the hydrogel had a higher storage modulus (G′) than the loss modulus (G″) at low strains, indicating its viscoelastic colloid nature. As strain increased, the hydrogel transitioned from a gel to a quasi-liquid state when the applied strain exceeded 4.9%. Thehydrogel exhibited excellent self-healing properties, with immediate recovery of G′ and G″ observed upon repeated high (200%) and low (1%) strain cycles at a 1-Hz frequency (Fig. 3G, H). In vitro studies on the ROS-responsive degradation behaviour of the hydrogel revealed that hydrogel degradation was continually observed for 21 days. According to the findings, in a PBS environment at 37 °C, the hydrogel degraded approximately 50% on day 17. In the presence of 1 mM H_2_O_2_, the hydrogel degraded approximately 50% on day 7, with an increased degradation rate as the volume of the hydrogel decreased and the relative surface area increased. The degradation rate in the presence of 1 mM H_2_O_2_ was significantly higher than that with PBS (Fig. 3I). Similarly, MVs release from the hydrogel over the 21-day period was faster and in greater quantities in the presence of 1 mM H_2_O_2_, matching degradation the degradation rate of the hydrogel (Fig. 3J, K). These experiments collectively demonstrated the ROS responsiveness of the hydrogel synthesised with a ROS-Linker, which delayed MVs release and extended their half-life. The dynamic bonds allowed the hydrogel to recover its integrity after fracturing, particularly after injection into the knee joint, where shear and compressive forces can cause hydrogel dispersion. Rapid self-healing prevented excessive fragmentation of the hydrogel, leading to premature MVs release and degradation [59]. In addition, the dynamic bonds were less likely to recover in areas with high local ROS concentrations, promoting local MVs release and achieving intelligent anti-cell ageing.

### Biocompatibility of the hydrogel

**Fig. 4:**
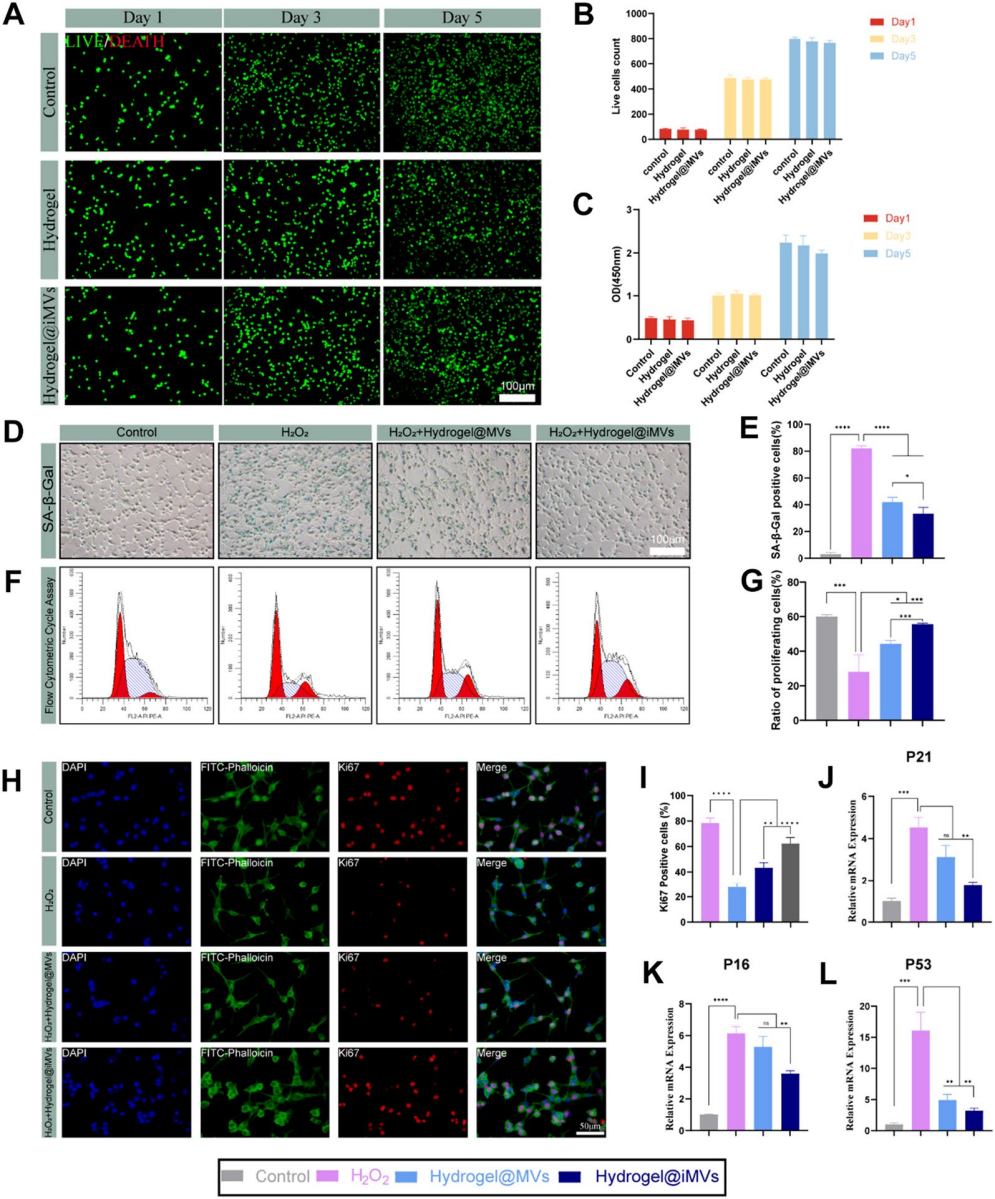
In vitro cell compatibility and anti-aging effects of Hydrogels@MVs/iMVs. **A**, **B** Live (green)/dead (red) fluorescence results and live cell counts of Control, Hydrogel, and Hydrogel@MVs groups on day 1, 3, and 5. **C** CCK-8 results of Control, Hydrogel, and Hydrogel@iMVs groups on day 1, 3, and 5. **D**, **E** SA-β-Gal staining and percentage of senescent cells after ageing induction and intervention with different factors. **F**, **G** Cell cycle analysis and percentage of proliferating cells after the same treatment using flow cytometry. **H**, **I** Immunofluorescence experiments were conducted to assess the Ki67 positivity rate in cells after treatment, followed by statistical analysis. **J**–**L** RT-PCR analysis of the relative expression levels of senescence markers P16, P21, and P53 mRNA in the different treated groups. (*P < 0.05, **P < 0.01, ***P < 0.001, ****P < 0.0001, n = 3)

We established a co-culture model of hydrogel and chondrocytes using the Transwell apparatus to assess the impact of the hydrogel on the normal growth of chondrocytes. ATDC5 cells were co-cultured with the hydrogel for 5 days and cell viability was analysed on days 1, 3, and 5. Since MVs/iMVs are normal secretory components of MSCs, and previous experiments confirmed their lack of impact on cell viability, biocompatibility experiments were conducted on the Control, Hydrogel, and Hydrogel@iMVs groups. The results of live/dead cell staining showed a small number of dead cells (indicated by red fluorescence) in the Hydrogel and Hydrogel@iMVs groups on days 3 and 5, but no statistically significant change was observed compared to the Control group (Fig. 4A, B). Furthermore, the CCK-8 experiment confirmed that the hydrogel had no discernible impact on cell growth (Fig. 4C). These tests demonstrated that the hydrogel is highly biocompatible and does not adversely affect cell viability.

### Therapeutic effects on ATDC5 cell senescence

To evaluate the therapeutic effects of MVs/iMVs on chondrocyte senescence in vitro, we induced ATDC5 cell senescence using H_2_O_2_ and separately treated them with Hydrogel@MVs/iMVs.Senescent cells exhibit β-galactosidase activity, allowing us to perform SA-β-Gal staining to count the number of positive cells. Results indicated that after H_2_O_2_ induction, approximately 80% of ATDC5 cells exhibited significant senescence. However, treatment with MVs/iMVs reduced the proportion of senescent cells to around 40%. Notably, the anti-senescence effect of Hydrogel@iMVs was significantly superior to that of Hydrogel@MVs (P < 0.05) (Fig. 4D, E). Cell cycle arrest is a hallmark of cellular senescence. To further assess cell senescence, we conducted cell cycle analysis on cells subjected to the same conditions. The analysis revealed that H_2_O_2_ induction led to cell cycle arrest, with a decrease in the number of cells in the proliferation phase. The anti-senescence effect of Hydrogel@iMVs on cell cycle and cell senescence and was more pronounced than that of Hydrogel@MVs (P < 0.05) (Fig. 4F, G). To address potential limitations in detecting senescence using SA-β-Gal staining, we assessed Ki67 positivity in cells after treatment through immunofluorescent experiments [60]. Ki67 is a marker for proliferating cells and is not expressed in senescent cells. The results were consistent with previous cell cycle experiments. In the control group, approximately 80% of cells were Ki67-positive, while Ki67 positivity significantly decreased to approximately 30% in senescent cells. After treatment with Hydrogel@iMVs, Ki67 positivity markedly increased, with the treatment effect surpassing that of Hydrogel@MVs (P < 0.05) (Fig. 4H, I). In ATDC5 cells subjected to various treatments, RT-PCR analysis detected the expression of P16, P21, and P53. The expression of these genes in the Hydrogel@iMVs group was significantly lower than that in the senescence group, and only the expression of P53 in the Hydrogel@MVs group significantly differed from that in the H_2_O_2_ group (P < 0.05) (Fig. 4J–L). Additionally, we assessed the expression levels of both RB protein and p-RPS6 protein in each group, and the results were consistent with all experimental findings (Additional file 1: Fig. S4). Therefore, our findings suggest that iMVs may have better effects than MVs in combating cellular senescence.

### Improvement in cellular energy metabolism

**Fig. 5:**
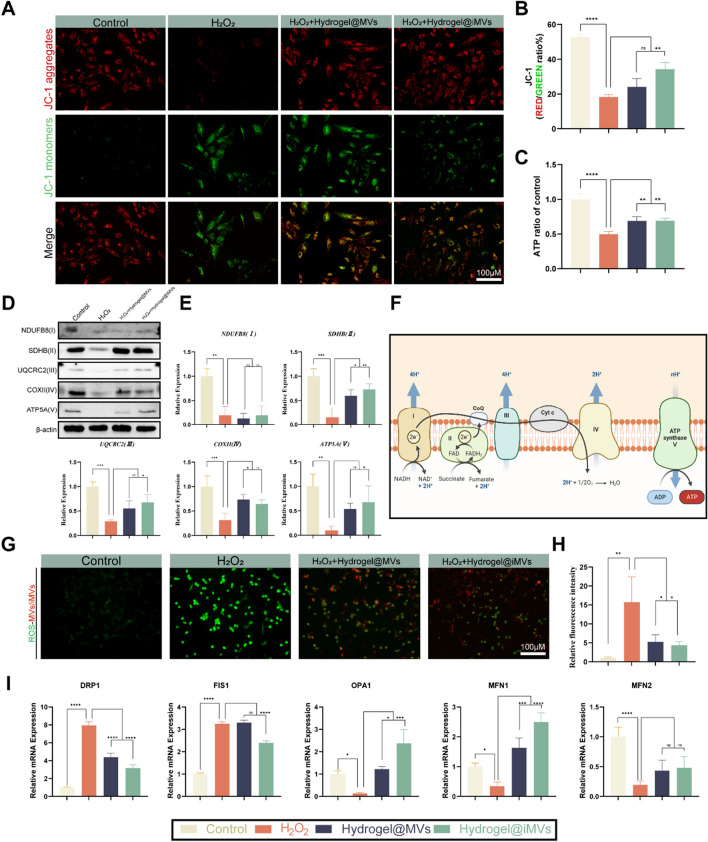
The effects of Hydrogel@MVs/iMVs on mitochondrial function and anti-oxidative stress in ATDC5 cells in vitro. **A**, **B** Analysis of mitochondrial membrane potential in different treatment groups using JC-1 staining. Red represents JC-1 aggregates with high mitochondrial membrane potential, and green represents JC-1 monomers with low mitochondrial membrane potential. The ratio of red to green fluorescence was calculated for each group. **C** Detection and quantification of ATP in each group. **D**, **E** Relative expression levels of respiratory chain complexes (I–V) in the mitochondrial inner membrane were detected by Western blotting. **F** Schematic diagram of the mitochondrial inner membrane respiratory chain. **G**, **H** The residual level of ROS in cells was detected by the ROS probe DCFH-DA, and the relative fluorescence intensity of the probe was analyzed to reflect the anti-oxidative capacity. **I** RT-PCR was used to detect the relative mRNA expression levels of mitochondrial fission genes DRP1, FIS1, and mitochondrial fusion genes OPA1, MFN1, and MFN2 in each treatment group. (*P < 0.05, **P < 0.01, ***P < 0.001, ****P < 0.0001, n = 3)

ATP generation and mitochondrial membrane potential are crucial markers for assessing mitochondrial function, directly linked to cellular senescence [61]. Increasing mitochondrial membrane potential can effectively combat cellular senescence [13, 62]. We hypothesised that Hydrogel@MVs/iMVs might exert their therapeutic effects by improving mitochondrial function. Therefore, we employed JC-1 staining to assess mitochondrial membrane potential in senescent ATDC5 cells. The results indicated a significant increase in green fluorescence, representing low mitochondrial membrane potential, and a notable decrease in red fluorescence, representing high mitochondrial membrane potential, in senescent cells. After the addition of Hydrogel@ MVs/iMVs, the mitochondrial potential was restored. The red-to-green fluorescence ratio indicated a substantial improvement in mitochondrial membrane potential, particularly in the Hydrogel@iMVs group, whereas there was no significant difference in the Hydrogel@ MVs group (P < 0.05) (Fig. 5A, B). ATP detection further revealed that ATP production in ATDC5 cells significantly improved after treatment with Hydrogel@ MVs/iMVs compared to the ageing group (Fig. 5C). To delve into the mechanisms behind the improved mitochondrial function, we analysed the mitochondrial respiratory chain complexes (I–V) using Western Blotting. Our findings indicated that treatment with MVs led to varying degrees of improvement in these respiratory chain complexes. Hydrogel@MVs had a more pronounced effect on complexes II and IV, while Hydrogel@iMVs had a significant impact on complexes II, III, and V (P < 0.05) (Fig. 5D, E). The respiratory chain complexes are essential functional proteins located in the mitochondrial inner membrane, and their decline is a major contributor to mitochondrial dysfunction, which can lead to an increase in ROS (Fig. 5F).

### Anti ROS function evaluation

ROS plays a pivotal role in linking cell ageing and mitochondrial dysfunction, making it a critical aspect of cell ageing treatment. Excessive ROS not only damages cellular DNA and protein structures but is also released into the extracellular environment, leading to elevated ROS levels in the microenvironment [63]. Thus, improving the cell’s resistance to ROS is key to breaking the vicious cycle of cell cellular ageing. To evaluate the effects of Hydrogel@MVs/iMVs, we exposed cells to ROS in normal culture medium for 48 h, replaced it with fresh complete medium, and subsequently introduced Hydrogel@MVs/iMVs carrying PKH26 fluorescent probes for 24 h. Using the ROS probe DCFH-DA, we measured residual levels of ROS within the cells and analysed the relative fluorescence intensity of the probe. The results showed that intracellular ROS levels in cells treated with Hydrogel@MVs/iMVs significantly decreased. Notably, cells with higher levels of red fluorescence, indicating higher engulfment of MVs/iMVs, exhibited lower intracellular ROS levels (P < 0.05) (Fig. 5G, H). The cell’s resistance to ROS may be attributed to the regulation of mitochondrial movement and the promotion of mitochondrial fusion by MVs/iMVs. Through this fusion, mitochondria can withstand and improve resilience in high ROS environments while inhibiting excessive mitochondrial fragmentation [64–66]. Therefore, we used RT-PCR to assess the expression of genes associated with mitochondrial fission and fusion. Among these, DRP1 and FIS1 promote mitochondrial fission and fragmentation, while OPA1, MFN1, and MFN2 promote mitochondrial fusion. The results indicated that both types of MVs had a certain degree of promoting mitochondrial fusion. However, iMVs exhibited superior performance in inhibiting fission and promoting fusion compared to MVs (P < 0.05) (Fig. 5I).

### Therapeutic effect on osteoarthritis in vivo

**Fig. 6:**
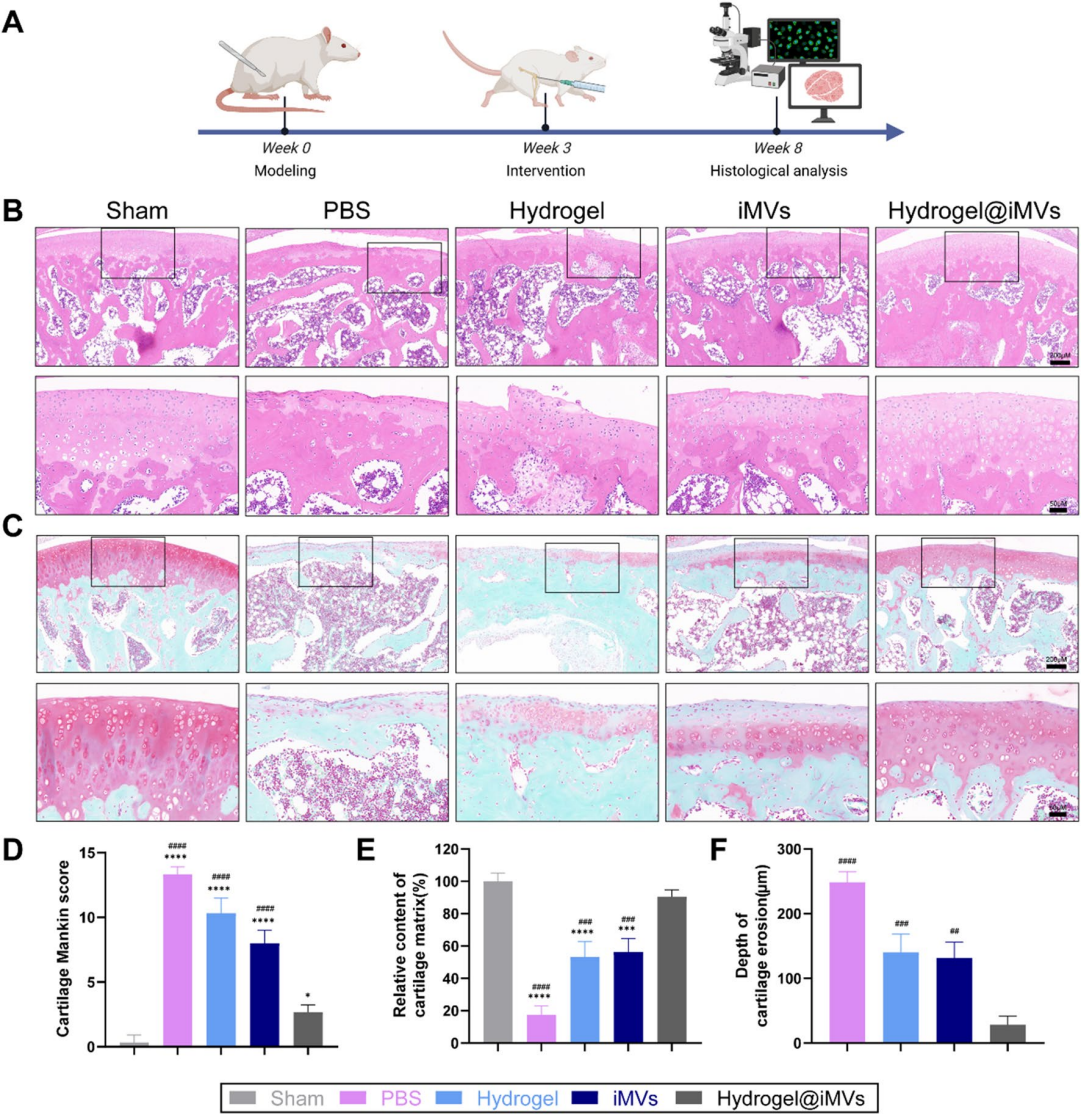
Histological staining. **A** Schematic diagram of animal modeling and intervention time. **B**, **C** Representative images of knee joint sections from each group stained with H&E and Safranin O-Fast Green. **D**–**F** Modified Mankin scores, relative content of cartilage matrix, and depth of cartilage erosion in each group. (*and #indicate comparison with sham group and Hydrogel@iMVs group, *P < 0.05, **P < 0.01, ***P < 0.001, ****P < 0.0001, #P < 0.05, ##P < 0.01, ###P < 0.001, ####P < 0.0001, n = 5)

Following in vitro experiments that demonstrated the superior effectiveness of iMVs over MVs in inhibiting cellular senescence and enhancing mitochondrial function, we selected iMVs for subsequent animal studies to assess the in vivo therapeutic effects of Hydrogel@iMVs. After an 8-week modelling period with 5 weeks of intervention, the rats were euthanised, and histological analyses were performed (Fig. 6A). Knee joints were also subjected to Safranin O-fast green and H&E staining to assess histological changes on the cartilage surface (Fig. 6B, C). Observations indicated that the cartilage surface in the sham group remained smooth with a normal structure, featuring a thick cartilage layer and a normal cell count. In contrast, the PBS group displayed severe degenerative changes in the joint cartilage, with a severely worn cartilage surface and disordered cartilage cells. Additionally, Safranin O-fast green staining was faint. In the Hydrogel@iMVs group, the cartilage surface exhibited only slight wear, with no significant degeneration. These findings confirmed the therapeutic efficacy of Hydrogel@iMVs, as supported by Mankin score analysis [67]. Furthermore, cartilage erosion and relative matrix content were evaluated (Fig. 6D–F). The results demonstrated that compared with the PBS group, the empty hydrogel group and the single iMVs injection group had a certain degree of therapeutic effect. However, varying degrees of degeneration and wear were still observed. This underscores the importance of the responsive hydrogel in extending the action time of iMVs and thereby enhancing the therapeutic effect (P < 0.05). Immunohistochemical analysis of ageing markers was conducted, revealing that the cartilage cells in the PBS group exhibited evident signs of ageing, whereas the Hydrogel@iMVs group significantly improved cartilage cell (Additional file 1: Fig. S3).

**Fig. 7:**
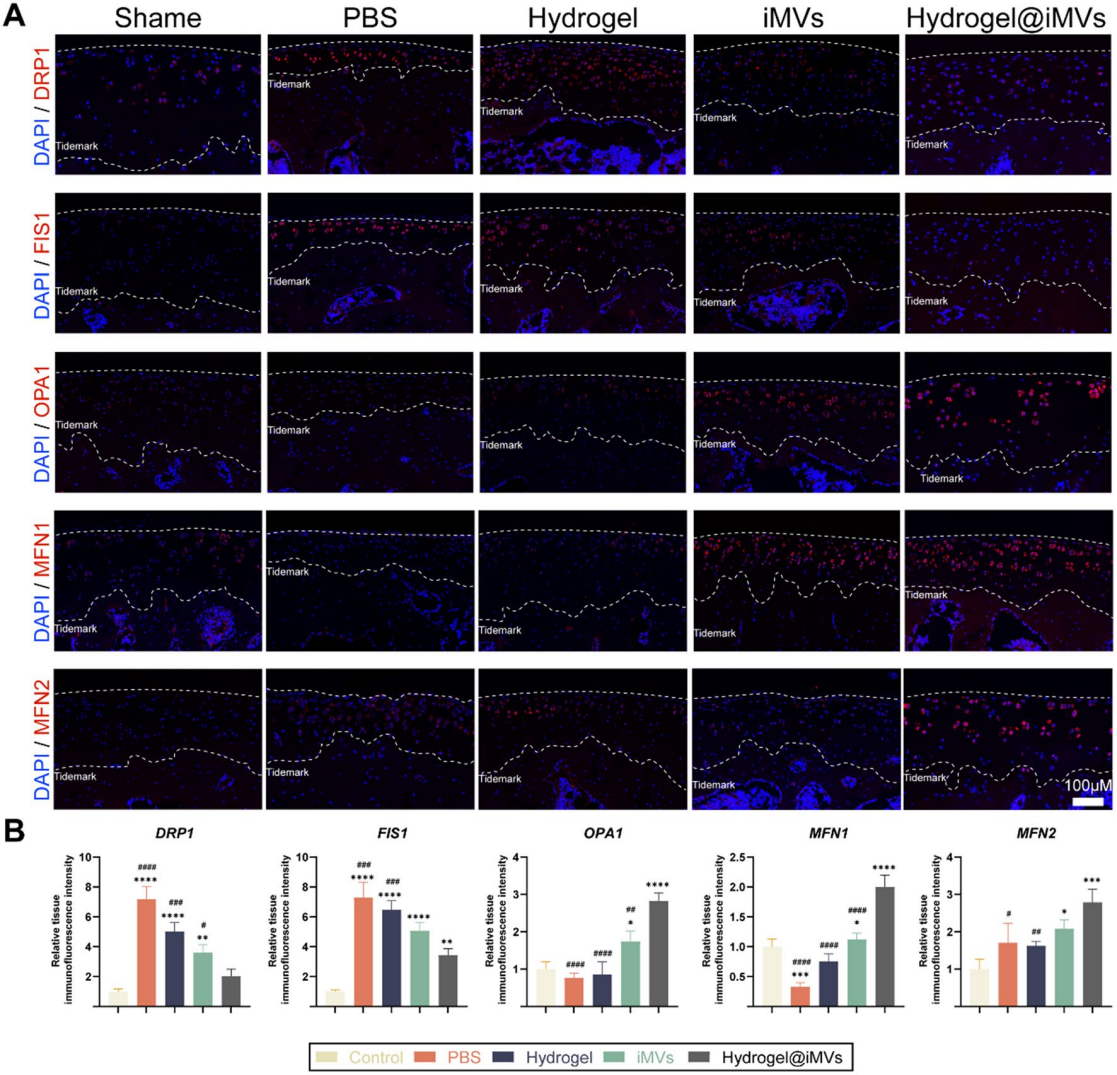
Tissue immunofluorescence staining. **A**, **B** Relative expression levels of mitochondrial fission and fusion proteins in each group were analyzed by immunofluorescence staining (*and #compared with sham group and Hydrogel@iMVs group, *P < 0.05, **P < 0.01, ***P < 0.001, ****P < 0.0001, #P < 0.05, ##P < 0.01, ###P < 0.001, ####P < 0.0001, n = 5)

Additionally, immunofluorescence experiments on knee joint tissue slices were performed, analysing the expression of mitochondrial fusion proteins OPA, MFN1, MFN2, and mitochondrial fission proteins DRP1 and FIS1 in cartilage cells and conducting relative fluorescence intensity analysis (Fig. 7A, B). The results indicated that in OA, the expression level of the fission protein in joint cartilage cells significantly increased, while the expression of fusion proteins markedly decreased. However, following treatment with Hydrogel@iMVs, DRP1, and FIS1 decreased, while OPA1 and MFN1 exhibited a significant upward trend, and MFN2 showed a slight increase. These findings further validate the therapeutic efficacy of the material in improving mitochondrial function in vivo (P < 0.05). In summary, Hydrogel@iMVs effectively inhibits cartilage cell wear and delays the progression of OA by enhancing mitochondrial function and breaking the vicious cycle of senescence.

## Discussion

OA is a degenerative joint disease that is closely associated with ageing, and its incidence continues to rise with advancing age. Several reports highlight the pivotal role of senescent chondrocytes in the onset and progression of OA. It has been demonstrated that the accumulation of senescent chondrocytes is intricately linked to mitochondrial dysfunction and the resulting excessive accumulation of ROS [68]. Mitochondrial dysfunction is a significant hallmark of cellular ageing, characterised by excessive mitochondrial fission and vacuolisation in ageing chondrocytes, leading to increased ROS release, decreased membrane potential, reduced ATP levels, and impaired mitochondrial complexes. The increase in ROS resulting from mitochondrial dysfunction further exacerbates the impairment of normal mitochondria [69]. Meanwhile, excessive ROS are released into the extracellular environment, accelerating the progression of OA [70]. Current approaches to alleviate ageing-related diseases primarily involve selectively eliminating senescent cells to achieve therapeutic benefits [71]. In the case of OA, excessive removal of cells can disrupt normal tissue structure, particularly considering that cartilage is predominantly composed of chondrocytes, and these cells are relatively sparse. Thus, a more advantageous approach is to improve the senescence of chondrocytes.

Stem cells have demonstrated significant potential in addressing cellular senescence [72]. It is widely acknowledged that the therapeutic effects of stem cells are mainly mediated through paracrine signalling, with extracellular vesicles, particularly MVs, being key components. MVs play a pivotal role in intercellular communication, modulating inflammation, immune responses, and cellular metabolism, serving as vital mediators within the paracrine machinery. Therefore, this study focused on MSCs-derived MVs. Nevertheless, MVs have a limited half-life and lack responsiveness to the ROS-rich environment [73]. ROS, with its continuous accumulation in chondrocytes and the local OA microenvironment, initiates a vicious cycle of ageing, representing a pivotal factor in the progression of both OA and chondrocyte senescence [74]. Hence, effectively responding to the ROS-enriched environment is crucial for enhancing the efficacy of MVs. To address these limitations, an injectable ROS-responsive hydrogel loaded with MVs was developed. It exhibited good biocompatibility and injectability, minimising the trauma associated with drug administration. Furthermore, Hydrogel@iMVs could respond to ROS, releasing MVs more effectively when local ROS concentrations were elevated, thereby achieving on-demand release and enhancing the efficacy of iMVs.

Additionally, using cytokines or other bioactive substances as a pre-stimulation to generate MVs is gaining increasing attention. In a normal environment, MSCs remain in a quiescent state, characterised by an unstable and heterogeneous secretion profile. When exposed to cytokine stimulation, they enhance the production of regulatory molecules, which become enriched in MVs while also reducing MV’s heterogeneity [75]. Therefore, MSCs may produce MVs with varying therapeutic effects under different pre-stimulation conditions. IFN-γ, an ageing-related cytokine, serves as a crucial inflammatory mediator and plays a role in immune regulation, antiviral defence, and anti-tumour activity [76]. Many immune and inflammation-regulating cells in the body can produce IFN-γ, and its receptor IFNGR1 is widely expressed in most cells, modulating their functions. MSCs are among the cell types subject to this regulation, and IFN-γ is one of the primary cytokines involved in MSC activation [77, 78]. Given that OA is an inflammatory and age-related condition, using IFN-γ as a ‘trigger’ for pre-stimulation in generating MSC-derived MVs may lead to superior effects in ameliorating chondrocyte senescence. In our in vitro experiments, hydrogel@iMVs exhibited a superior ability to reduce ROS production, improve mitochondrial function, and correct cellular senescence phenotypes. This highlights that IFN-γ, as a stimulating factor, can effectively enhance the therapeutic effects of MVs hydrogels.

In vivo experiments further confirmed the excellent therapeutic potential of Hydrogel@iMVs in the context of OA. We confirmed through tissue immunofluorescence and immunohistochemistry that intra-articular injection of Hydrogel@iMVs in a rat model of OA effectively regulates the balance of mitochondrial fusion and fission proteins, ultimately improving chondrocyte senescence. Additionally, its therapeutic effect is superior to that of the pure material group and the iMVs group. In OA, chondrocytes, as the sole cell type within cartilage, play a pivotal role in determining the fate of the cartilage. Our findings from H&E staining and Safranin O-fast green staining revealed that by maintaining the normal state of chondrocytes, it is possible to effectively preserve the thickness of the cartilage matrix, reduce erosion and degeneration of the cartilage layer, and maintain the balance of cartilage synthesis and degradation metabolism within the body [74]. In summary, both in vitro and in vivo experiments demonstrate that our innovative ROS-responsive Hydrogel@iMVs showed the ability to enhance mitochondrial function, improving chondrocyte senescence, and significantly alleviate early-stage cartilage degeneration caused by OA.

## Conclusion

In summary, the present study introduces a promising ROS-responsive hydrogel loaded with IFN-γ-pre-stimulated iMVs for the treatment of OA. This approach aims to enhance mitochondrial function in chondrocytes through the intelligent release of iMVs, effectively ameliorating chondrocyte senescence and inhibiting cartilage degeneration. The hydrogel exhibits injectability, self-healing capability, and ROS-responsive properties due to the dynamic bonds formed between PBA grafted on SF and neighbouring diols in PVA. It also extends the duration of iMVs’ effectiveness, thereby enhancing its therapeutic efficacy. Our in vitro experiments validate the excellent biocompatibility of the hydrogel, while Hydrogel@iMVs surpasses hydrogel@MVs in promoting mitochondrial function and anti-ageing properties. Subsequent animal experiments further establish that Hydrogel@iMVs promotes the expression of mitochondrial fusion proteins, inhibits excessive fission, and effectively ameliorates chondrocyte senescence and cartilage degeneration. This novel ROS-responsive iMVs-loaded hydrogel system presents a promising approach for mitigating chondrocyte senescence in OA.

### Supplementary Information

**Additional file 1:** **Figure S1.** Screening for H_2_O_2_ Concentrations Inducing Cellular Senescence. **Figure S2.** MVs SEM in 20-day immersion solution of Hydrogel.** Figure S3.** Expression of senescence hallmark in chondrocytes in vivo experiments. **Figure S4.** Observation of mitochondrial morphology in ATDC5 cells, in vitro experiments. **Figure S5.** Detection of cell senescence-related proteins in ATDC5 Cells. **Table S1.** Primers sequence of each gene in the experiment.

## Data Availability

The data and material are available.

## Abbreviations

OA: Osteoarthritis
ROS: Reactive oxygen species
MVs: Microvesicles
IFN-γ: Interferon-gamma
APBA: 3-Aminophenylboronic acid
SF: Silk fibroin
PVA: Polyvinyl alcohol
SASP: Senescence-associated secretory phenotype
MSCs: Mesenchymal stem cells
EDC: N-(3-Dimethylaminopropyl)-n′-ethylcarbodiimide hydrochloride
NHS: N-hydroxysuccinimide
2-ME: 2-Mercaptoethanol
DMEM: Dulbecco’s modified eagle medium
FBS: Fetal bovine serum
PBS: Phosphate buffered saline
SA-β-Gal: Senescence-associated β-galactosidase
SEM: Scanning electron microscopy
ACLT: Anterior cruciate ligament transection
DMM: Destabilizing the medial meniscus
IACUC: Institutional Animal Care and Use Committee
ANOVA: One-way analysis of variance
