# Supplementary material for: Microvesicles-hydrogel breaks the cycle of cellular senescence by improving mitochondrial function to treat osteoarthritis
Source: J Nanobiotechnology. 2023 Nov 15;21:429. doi: 10.1186/s12951-023-02211-8 (PMC10652587; doi:10.1186/s12951-023-02211-8)
Supplement: Supplementary file 1 — Additional file 1: Figure S1. Screening for H2O2 Concentrations Inducing Cellular Senescence. Figure S2. MVs SEM in 20-day immersion solution of Hydrogel. Figure S3. Expression of senescence hallmark in chondrocytes in vivo experiments. Figure S4. Observation of mitochondrial morphology in ATDC5 cells, in vitro experiments. Figure S5. Detection of cell senescence-related proteins in ATDC5 Cells. Table S1. Primers sequence of each gene in the experiment. [file 12951_2023_2211_MOESM1_ESM.docx]

**Microvesicles-Hydrogel breaks the cycle of cellular senescence by improving mitochondrial function to treat osteoarthritis**

*Senrui Liu*^1^*, Shengwen Cheng*^1^*, Bowen Chen*^1^*, Pengcheng Xiao*^1^*,* *Jingdi Zhan*^1^*, Jiacheng Liu*^1^*, Zhuolin Chen*^1^*, Junyan Liu*^1^*, Tao Zhang*^1^*, Yiting Lei*^1^**, Wei Huang*^1^***

^1^ Department of Orthopaedic Surgery, the First Affiliated Hospital of Chongqing Medical University, Chongqing, 400016, P.R. China

* E-mail: leiyit614@163.com

* E-mail: huangwei68@263.net

**
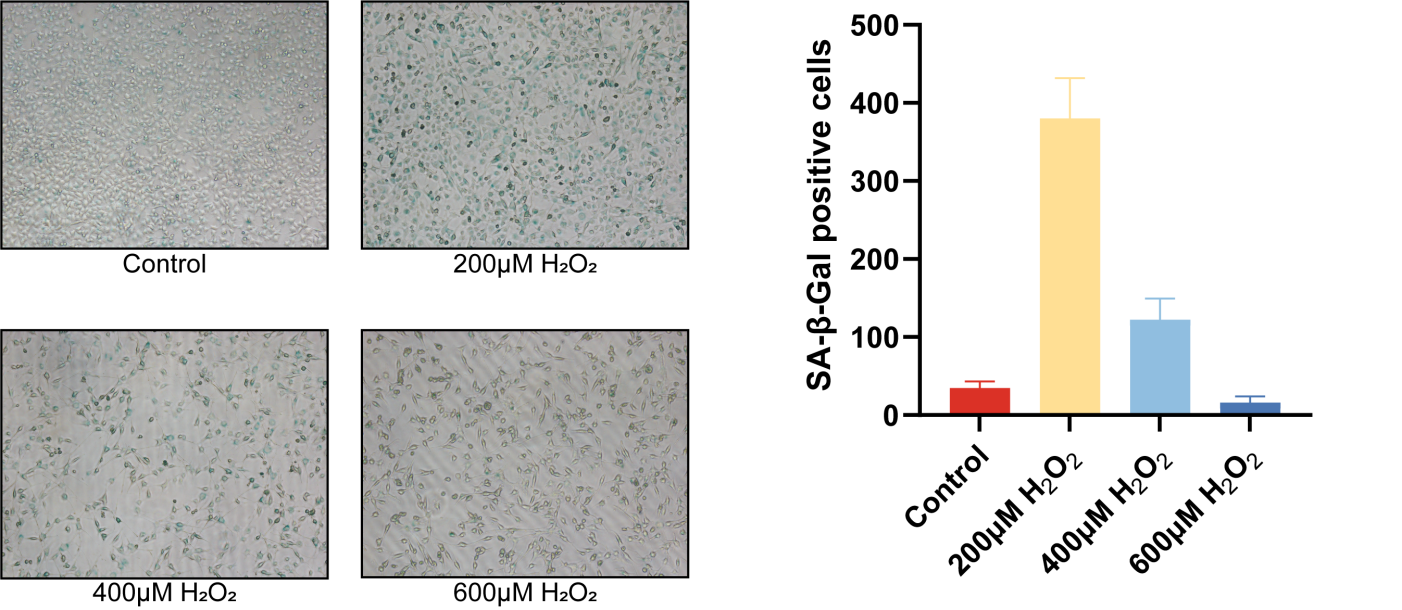
**

**Figure S1**: **Screening for H₂O₂ Concentrations Inducing Cellular Senescence.** SA-β-Gal staining and statistics of cellular senescence induced by different concentrations of H_2_O_2_.

**Figure S2:** **MVs SEM in 20-day immersion solution of Hydrogel.**


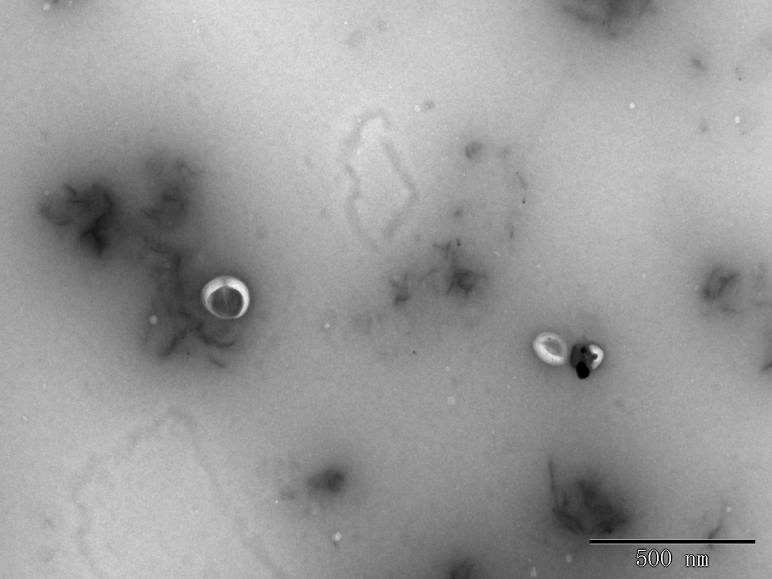

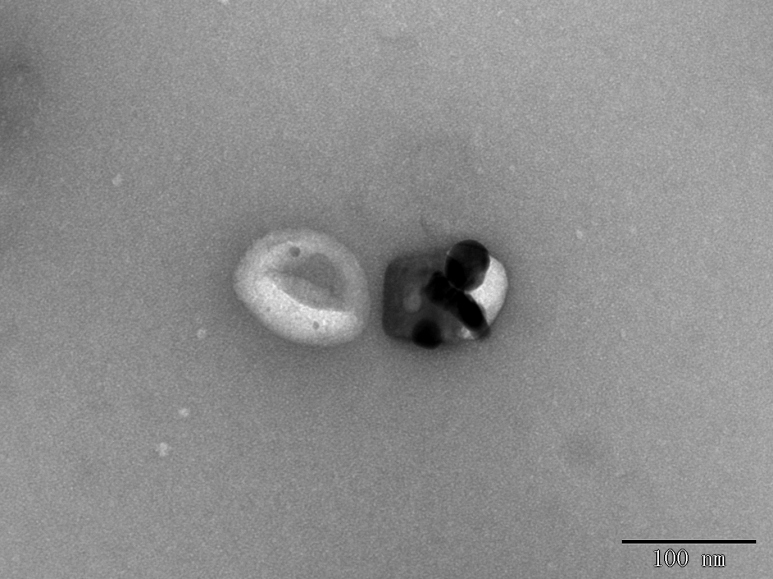


**Figure S3**: **Expression of senescence [hallmark](http://www.baidu.com/link?url=dbvzSm7KBzIwG2iAWawQmubAxHHTkJbM-JXPgwXWyhtEBOmCjBOnoFlaEbSPlDpu) in chondrocytes in vivo experiments.** Immunohistochemical staining and relative expression of P53 in different treatment groups in vivo. (* and # indicate comparison with sham group and Hydrogel+iExos group, *P < 0.05, **P < 0.01, ***P < 0.001, ****P < 0.0001, #P < 0.05, ##P <0 .01, ###P < 0.001, ####P < 0.0001, n=5).

**
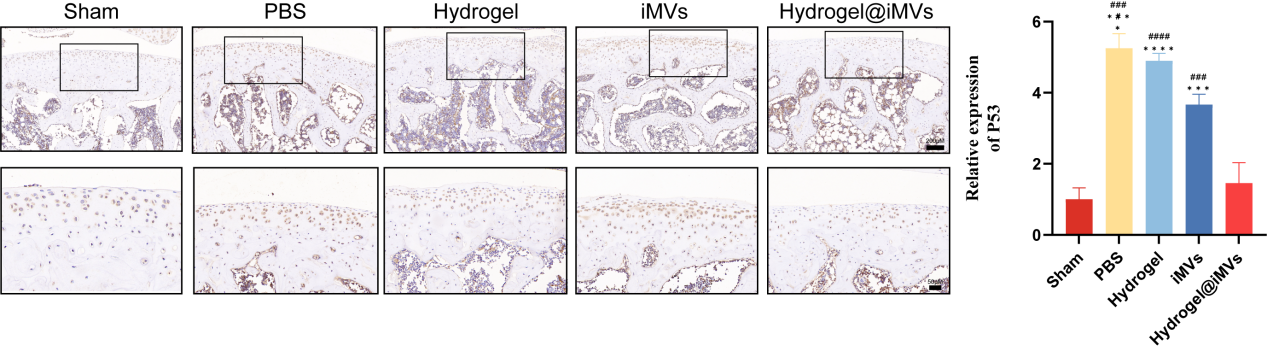
**

**Figure S4**: **Transmission Electron Microscopy Analysis of Mitochondrial Morphology and Dynamics in ATDC5 Cells.** ATDC5 cells were subjected to various treatments: H_2_O_2_, H_2_O_2_+Hydrogel@MVs treatment, and H_2_O_2_+Hydrogel@iMVs treatment. Control group received no specific treatment. Ultrastructural changes in mitochondrial morphology and dynamics were investigated using transmission electron microscopy, with a scale bar of 200 nm.


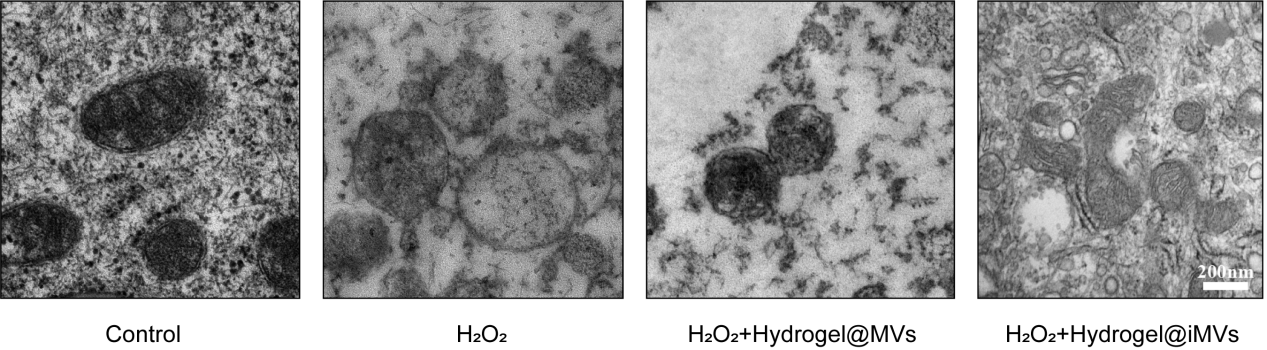


**Figure S5: Detection of cell senescence-related proteins in ATDC5 Cells.** Proteins were extracted from ATDC5 cells after different treatments, and Western blot experiments were conducted to detect RB and p-RPS6 proteins. Subsequently, a statistical analysis was performed on the relative expression levels (*P < 0.05, **P < 0.01, ***P < 0.001, ****P < 0.0001, n=3).


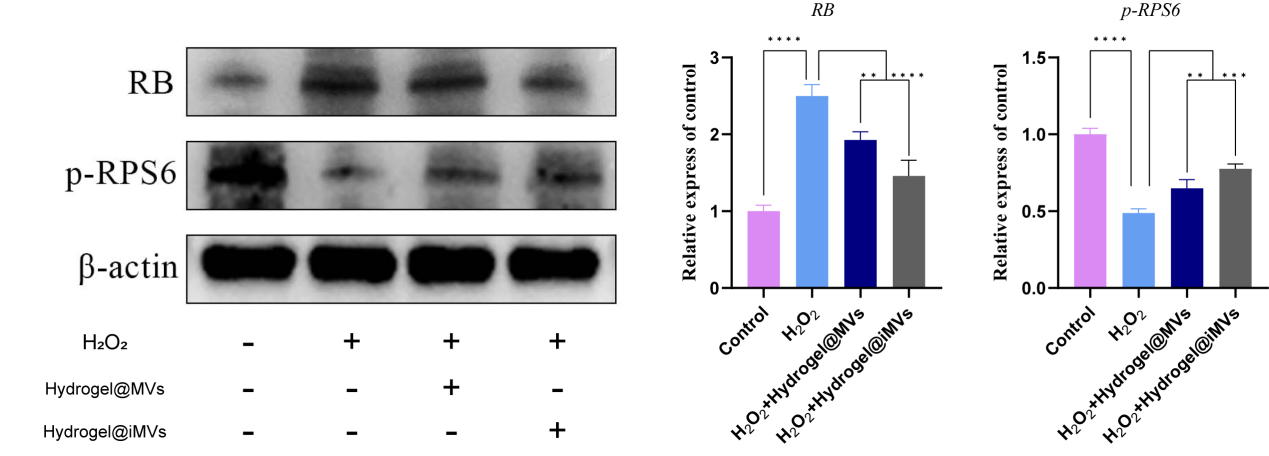


**Table S1: Primers sequence of each gene in the experiment.**

| Primer name | Orientation | Sequence (5´-3´) |
| --- | --- | --- |
| GAPDH | Forward  Reverse | CAGGAGAGTGTTTCCTCGTCC  TTTGCCGTGAGTGGAGTCAT |
| P16 | Forward  Reverse | CGTACCCCGATTCAGGTGATG  TCTGCACCGTAGTTGAGCAG |
| P21 | Forward  Reverse | AGAATAAAAGGTGCCACAGGC  AATCTGTCAGGCTGGTCTGC |
| P53 | Forward  Reverse | GGAAGACTCCAGTGGGAACC  CTTCTGTACGGCGGTCTCTC |
| FIS1 | Forward  Reverse | GAGACGAAGCTGCAAGGAATTT  CCAGGCACCAGGCATATTCA |
| DRP1 | Forward  Reverse | ACAGGAGAAGAAAATGGAGTTGA  CGTTGGGCGAGAAAACCTTG |
| OPA1 | Forward  Reverse | CTGCAGGTCCCAAATTGGTT  TCTTTGTCTGACACCTTCCTGT |
| MFN1 | Forward  Reverse | TGATCACAGGATTGGCGTCC  TGTTTTCCAAATCACGCCCC |
| MFN2 | Forward  Reverse | TGCCTGGATGCTGATGTGTT  AGAACTGCTTCTCCGTCTGC |
